# Paediatric subarachnoid haemorrhage and severe vasospasm secondary to traumatic pseudoaneurysm of a fenestrated vertebral artery: a case report and review of the literature

**DOI:** 10.1007/s00381-023-05894-4

**Published:** 2023-05-10

**Authors:** Michelle Kameda-Smith, Greg James, Kiran Seunarine, Adam Rennie, Fergus Robertson, Adikarige Haritha Dulanka Silva

**Affiliations:** 1 Great Ormond Street Hospital for Sick Children, London, England; 2 Department of Paediatric Neurosurgery, Great Ormond Street Hospital for Children, London, England; 3 Department of Radiology, Physics Group, London, England; 4 Paediatric Interventional Neuroradiology, London, England

**Keywords:** Vertebral artery fenestration, Paediatric traumatic aneurysm, Paediatric aneurysmal subarachnoid haemorrhage, Vasospasm

## Abstract

Paediatric intracranial aneurysms are rare entities accounting for less than 5% of all age intracranial aneurysms. Traumatic aneurysms are more common in children and have an association with anatomical variations such as arterial fenestrations. Here, we present a case of a child initially presenting with traumatic subarachnoid haemorrhage who returned to baseline and was discharged home only to return within 2 weeks with diffuse subarachnoid and intraventricular re-haemorrhage. A dissecting aneurysm of a duplicated (fenestrated) V4 vertebral artery segment was identified as a rare cause of rebleeding. We describe a course complicated by severe vasospasm delaying aneurysm detection and treatment. Dissecting aneurysms in children should be considered in all cases of delayed post-traumatic cranial rebleeding, particularly where there is anomalous arterial anatomy.

## Introduction

Paediatric intracranial aneurysms are rare, accounting for less than 5% of all age intracranial aneurysms [1–7]. A predilection for males is observed in young children with female preponderance occurring after puberty [1, 3, 8–11]. While a traumatic cause is rare in adult aneurysms (less than 1% of all age aneurysms) [12], traumatic aneurysms are more frequently observed in children, reported between 5 and 39% in paediatric series [10, 11, 13–16]. Anomalous intracranial arteries seem particularly vulnerable to aneurysm formation in otherwise insignificant trauma and post-traumatic aneurysms seem to be associated with significant morbidity and mortality [17].

Vertebral artery duplication (VAD) is a rare anomaly, with an incidence of 0.004–1.9%, often regarded as a vascular variant without clinical relevance [18–22]. Complete arterial duplications demonstrate dual origin of the vertebral artery (VA) from the ipsilateral subclavian artery or aortic arch. Vertebral artery fenestration (VAF) describes a partial duplication and are a rare vascular variant and observed in 2% of all intracranial artery fenestrations [23]. In contrast to true duplication, an anastomotic anomaly occurs during embryonic development resulting in a focal split of a vessel segment into two parallel channels which subsequently reconstitute a single vessel lumen. These anatomical variations have been observed both intra- and extracranially but occur most frequently at the V4 segment [21] and are frequently associated with other congenital intracranial vascular anomalies such as aneurysms and arteriovenous malformations [19, 23–33]. The mechanism for development of a V4 segment fenestration is the absence or obliteration of 2 intersegmental vessels that have fused [34], and its morphological changes may be responsible for its vulnerability to formation of aneurysm or development of arterial dissection. Specifically, histopathological examinations of fenestrated vessels have revealed irregularities in wall structure mainly involving the tunica media at the proximal and distal ends of the duplicated segments which can be less developed with an irregular pattern or complete absence of the elastic fibers [35, 36]. During severe cervical spine trauma involving rapid subluxation, deceleration or flexion through the cervical spine, the VA can be readily injured especially if irregularities in the vessels exist. To date, there have only been a handful of descriptions of this entity in the children (Table 1). However, we are the first to report a child developing a pseudoaneurysm in a VA fenestration after a significant head trauma. This case illustrates a need for special vigilance in managing children with vessel fenestrations or duplications in context of head trauma.

**Table 1:** Literature review of vertebral artery fenestration incidence and associated vascular anomalies

**Authors**	**Year**	**Number of vertebral angiographies**	**Number of vertebral fenestrations**	**Incidence**	**Associated anomaly**	**Identification**
Wollschlaeger et al. [18]	1967	291	1 (unknown)	0.34		Autopsy
Kowada et al. [19]	1972	362	5 adults 2 children	1.90	PComm aneurysm AComm aneurysm ECA-Vert anastomosis	Angiography
Carella et al. [20]	1978	1290	3 adults	0.23	Vertebral artery double fenestration	
Reiger et al. [25]	1983	500	2 adults	0.40		
Bharatha et al. [37]	2008	504	2 (unknown)	0.40	Aneurysm in 10.5% of all fenestrations (*n* = 53)	
Bayrak et al. [40]	2011	395	4 adults (54.32 + 15.3)	1.01	Saccular aneurysms in 14 patients	
D’Sa et al. [21]	2020	44,759	67 (2–95 years)	0.10	6 saccular aneurysms (9%) remote from site of fenestration involving ICA	
Omotoso et al. [22]	2021	554	2 (10–99 years)	0.004		

*AComm* anterior communicating, *PComm* posterior communicating, *ICA* internal carotid artery

## Case example

**Fig. 1:**
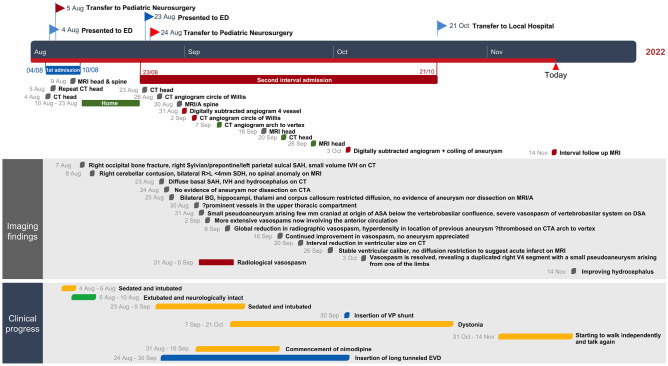
Clinical and radiological course post-subarachnoid haemorrhage

**Fig. 2:**
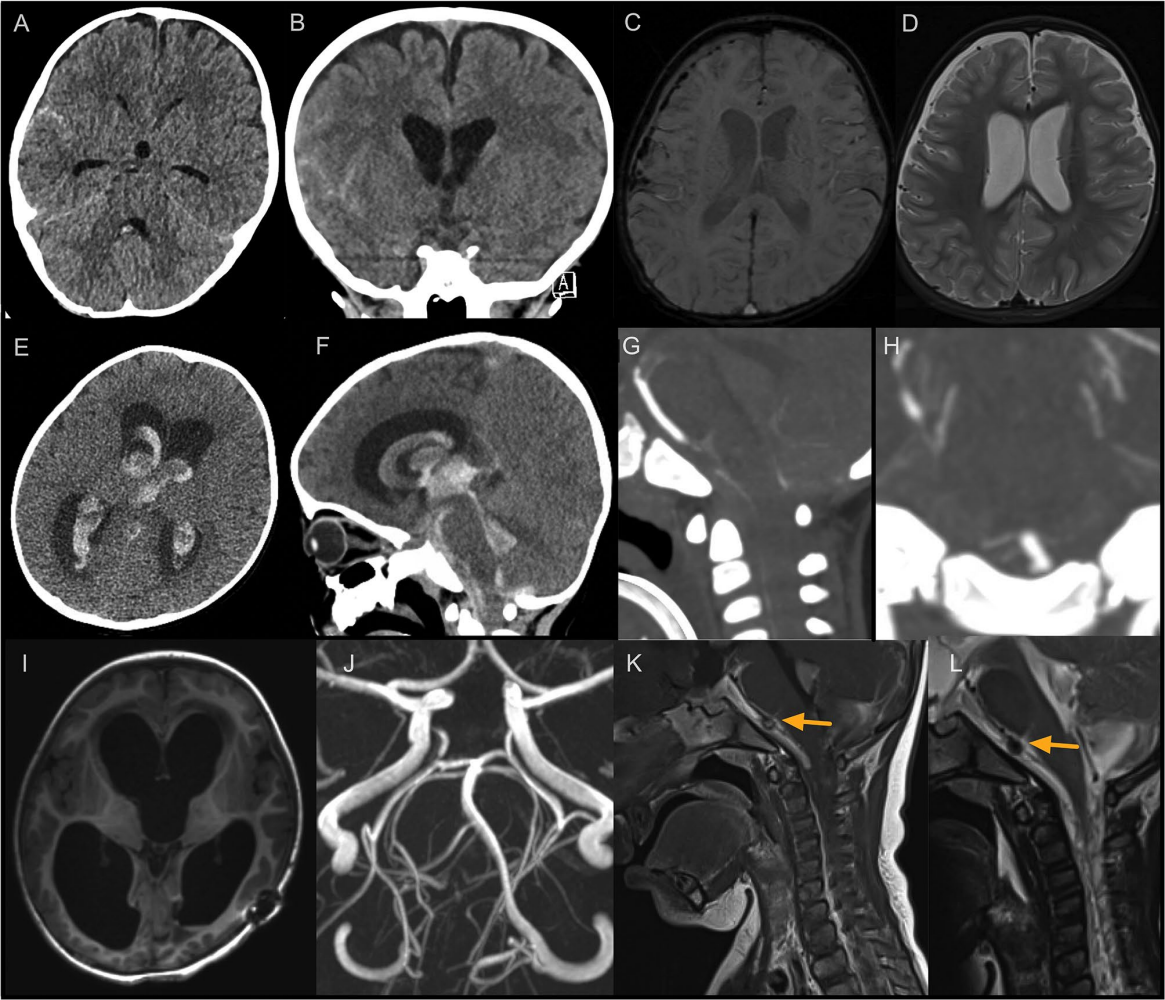
Radiological imaging of initial and interval presentation with investigations to identify aetiology of diffuse subarachnoid haemorrhage. **A–B** Initial neuroimaging post-traumatic head injury showing scattered SAH extending into the right Sylvian fissure and small volume intraventricular blood in the 4th ventricle. **C–D** MRI showing bilateral thin subdural hematomas. **E–F** Representation with acute deterioration and 4 ventricular IVH and hydrocephalus. **G–H** Initial CTA negative for vascular anomaly in the vertebrobasilar system. **I–J** hydrocephalus despite careful external ventricular drainage as no Aetiology of SAH found. **K–L** MRI/A identifying vertebral artery aneurysm (orange arrow)

A 14-month-old boy presented to his local hospital after a fall from his sibling’s arms while descending a flight of stairs followed by impaired consciousness (Fig. 1). On initial assessment, he was GCS 11–14 however with frequent fluctuations in conscious level and vomiting. He was intubated and CT head and neck scan identified an occipital skull fracture, scattered traumatic subarachnoid haemorrhage (SAH) and small volume dependent intraventricular haemorrhage (IVH) (Fig. 2A–B). He was neuro-protected for 24 h during which he was commenced on prophylactic levetiracetam for 7 days with a post-trauma MRI identifying no significantly concerning features (Fig. 2C–D). When weaned from ventilator and extubated, he was neurologically appropriate and therefore discharged home into the care of parents with paediatrician follow-up for head injury.

**Fig. 3:**
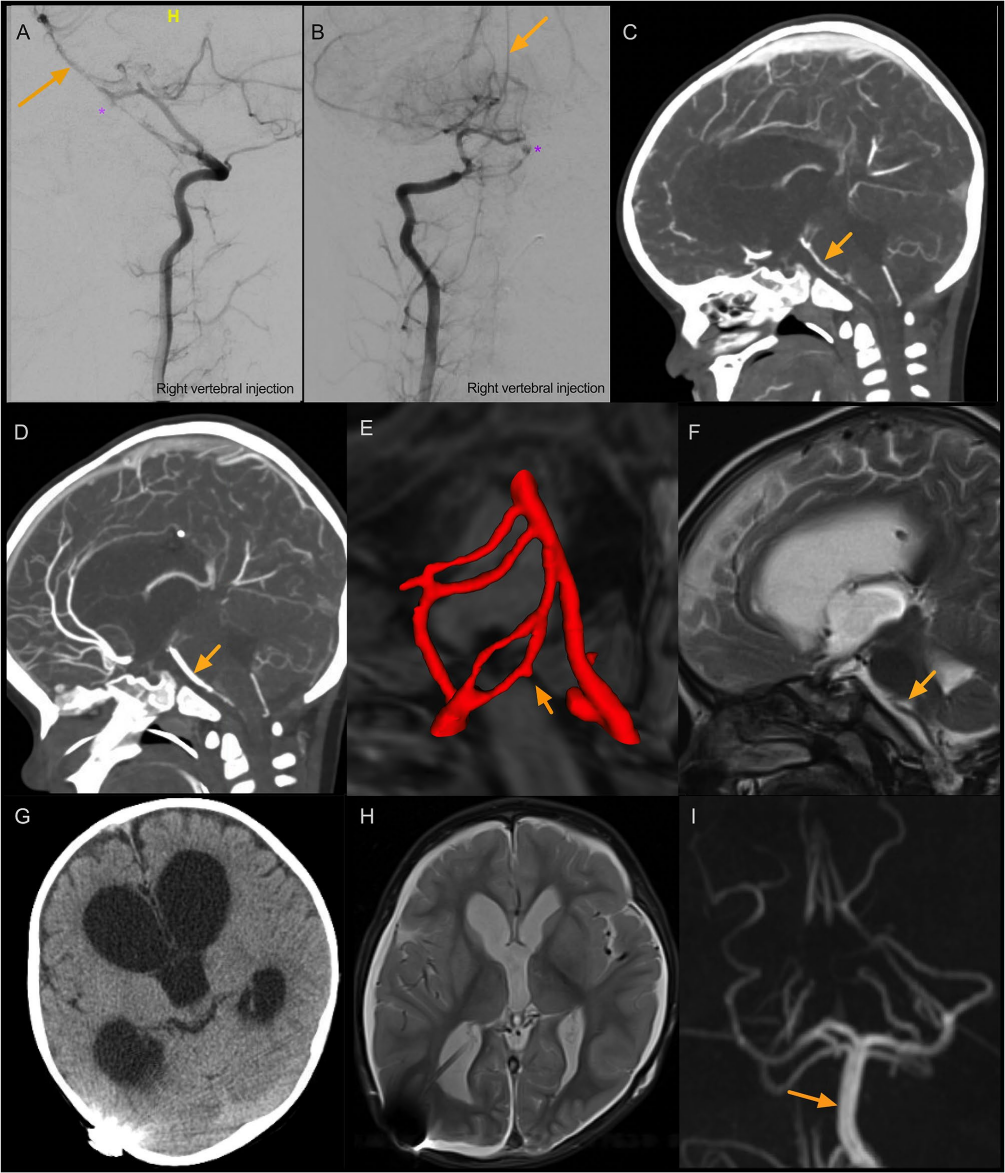
Identification of right intracranial V4 segment vertebral artery aneurysm and vasospasm course. **A–B** Right vertebral artery injection showing lateral and AP view of aneurysm (*) thought to arise from the left anterior spinal artery and severe vasospasm (orange arrow). **C** CTA 3 days later showing some more extensive vasospasm now involving the anterior circulation. **D** CTA 4 days later slowing some resolution of vasospasm. **E** CTA reconstruction 2 weeks later showing the aneurysm (orange arrow) on the medial arm of the V4 fenestration. **F** MRA 6 days later showing no aneurysm. **G** Pre-transfer CT showing maintained ventriculomegary. **H–I** Interval MRI showing reduction in ventricular calibre, and maintained calibre of posterior circulation vasculature (orange arrow)

**Fig. 4:**
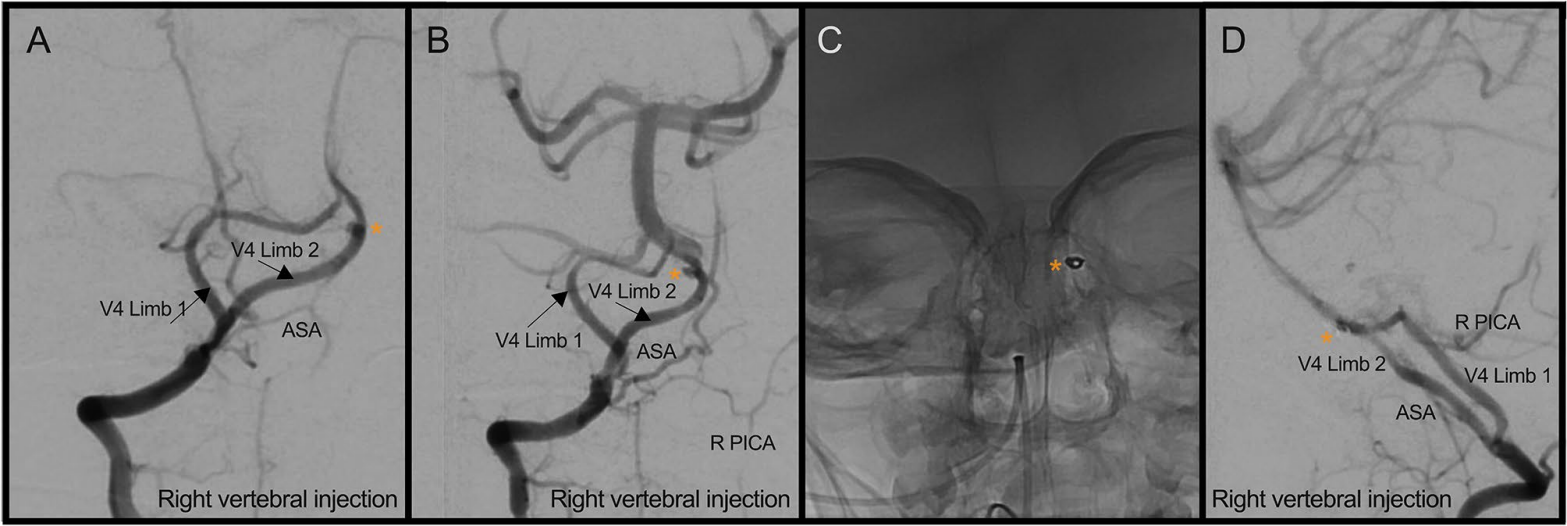
Definitive management of right V4 segment vertebral artery aneurysm after resolution of vasospasm revealing fenestration of artery associated with aneurysm. **A–B** Right vertebral injection showing oblique and AP views of vertebral artery fenestration at the V4 segment with aneurysm arising from the medial limb (*) only visible after vasospasm has resolved. **C–D** Coil embolization of aneurysm (*)

However, 20 days after his head injury, he became irritable in the afternoon with episodes of vomiting and holding his head in pain prompting re-admission to their local emergency department (ED) where he neurologically deteriorated to the point of respiratory support. CT imaging showed large volume intraventricular haemorrhage, hydrocephalus and diffuse subarachnoid haemorrhage extending into the cervical spine (Fig. 2E–F). He was urgently sent to a paediatric neurosurgical unit for external ventricular drain insertion and returned to the paediatric intensive care unit (PICU) for neuroprotection. Initial CT angiogram demonstrated severe cerebral vasospasm, but no vascular cause for SAH. MRI/A of the head and neck suggested a small aneurysm close to the vertebrobasilar junction (Fig. 2G–L). Digital subtraction catheter angiogram (DSA) confirmed the aneurysm however very severe vertebrobasilar artery vasospasm precluded definitive management of the lesion at this point (Fig. 3A–B). Over the course of 6 days, the radiological vasospasm improved though clinical status remained poor (Fig. 3C–D). With worsening radiographic ventriculomegaly and dystonia, the EVD was lowered to drain more CSF with an improvement in wakefulness. A ventriculo-peritoneal shunt using a MBlue valve (5/0) was inserted, and he continued to improve with re-emergence of speech, interaction with parents and improved dystonia. Cross-sectional imaging suggested interval resolution of the aneurysm with likely auto-thrombosis seen as hyperdensity on CT and absent flow void on MRI (Fig. 3E–F). Definitive repeat DSA after resolution of vasospasm identified the right vertebral pseudoaneurysm is now clearly arising from one arm of a distal V4 fenestration close to its confluence with the basilar artery (Fig. 4A–B). The aneurysm was embolized with a 2 mm × 2 cm EV3 axiom coil via a right common femoral arterial approach (Fig. 4C–D). Early follow-up MRI 1 month after transfer to local hospital for continued therapy showed improvement from the pre-transfer CT scan with maintained posterior circulation calibre (Fig. 3G–I).

## Discussion

While the history for our case is in keeping with a rupture of a post-traumatic pseudoaneurysm of the vertebral artery, subsequent identification of the aneurysm along the course of a V4 segment fenestration in a delayed interval angiogram calls into question whether this may have been a congenital aneurysm or congenitally vulnerable vessel to forming a post-traumatic aneurysm. Its location in the intracranial segment just after it pierces the dura at the lateral edge of the posterior atlanto-occipital membrane renders this region highly susceptible to acceleration-deceleration injury.

In patients presenting with a SAH and suspected ruptured aneurysm, arterial fenestrations of any vessels have been observed in up to 24%, with the majority observed in the anterior communicating artery (69%) [23]. Managing any ruptured aneurysm requires multidisciplinary discussion to evaluate both surgical and endovascular interventions, which can present specific challenges in vertebral arterial anomalies [37]. Surgical access to an aneurysm in the V4 segment is challenging due to complex local anatomy, including multiple perforators to the brain stem and lower cranial nerves. Endovascular treatment of aneurysms around the vertebrobasilar junction are now often first line for this reason. However, careful assessment of the angioarchitecture is required to establish arterial dominance, transarterial access to the aneurysm and amenability to vessel sacrifice, vessel remodelling or flow diversion often aided by three-dimensional imaging [38]. In this case, the fenestration of the right vertebral artery occurred distal to the ipsilateral posterior inferior cerebellar artery origin. The contralateral vertebral artery was dominant and did not fill the aneurysm. The cranial input to the anterior spinal axis arose from the fenestrated segment close to the aneurysm neck but was preserved following the coil embolization procedure. Furthermore, as the long-term natural history of our patient’s presumed pseudoaneurysm is unknown, non-invasive imaging and early catheter angiography is planned to carefully monitor for any progression.

The importance of continuing to search for the source of significantly diffuse SAH cannot be overemphasized. Our clinical case is an educational example of high clinical suspicion driving serial imaging, given the pattern of rebleeding and the high morbidity and mortality associated [39]. Furthermore, the paediatric group presents additional challenges due to smaller arterial calibre and lack of published evidence for various interventions considered common place in adult neurovascular management. A final point is the utilisation of a judicious approach in timing of intervention. In the aforementioned case, the resolution of spasm resulted in a far clearer understanding of the angioarchitecture of the morphology of the pseudoaneurysm and arterial circulation allowing a successful endovascular treatment option to be applied. Akin to other rare paediatric pathologies, children presenting with diffuse SAH should be managed as part of a multidisciplinary and specialized paediatric neurovascular team.

## Conclusion

Vertebral artery fenestrations are rare anatomical variants of normal arterial anatomy. However, in the setting of paediatric cranial trauma where development of traumatic intracranial aneurysms is proportionately higher than their adult counterpart, careful consideration in a multidisciplinary neurovascular team is vital.

## Data Availability

All data was available through EPIC and PACS systems for data and image acquisition.
